# Development of an open access systematic review instructional video series accessible through the SPI-Hub™ website

**DOI:** 10.5195/jmla.2025.2078

**Published:** 2025-01-14

**Authors:** Sheila V. Kusnoor, Annette M. Williams, Taneya Y. Koonce, Poppy A. Krump, Lori A. Harding, Jerry Zhao, John D. Clark, Nunzia B. Giuse

**Affiliations:** 1 sheila.v.kusnoor@vumc.org, Senior Information Scientist and Associate Director for Research, Center for Knowledge Management, Vanderbilt University Medical Center; Research Assistant Professor, Department of Biomedical Informatics, Vanderbilt University Medical Center, Nashville, TN; 2 Annette.williams@vumc.org, Senior Information Scientist and Associate Director for Metadata Management, Center for Knowledge Management, Vanderbilt University Medical Center, Nashville, TN; 3 taneya.koonce@vumc.org, Deputy Director, Center for Knowledge Management, Vanderbilt University Medical Center, Nashville, TN; 4 poppy.krump@vumc.org, Information Scientist, Center for Knowledge Management, Vanderbilt University Medical Center, Nashville, TN; 5 la.harding@vumc.org, Information Scientist, Center for Knowledge Management, Vanderbilt University Medical Center, Nashville, TN; 6 jerry.zhao@vumc.org, Senior Application Developer, Center for Knowledge Management, Vanderbilt University Medical Center, Nashville, TN; 7 john.clark@vumc.org, Center for Knowledge Management, Vanderbilt University Medical Center, Nashville, TN; 8 nunzia.giuse@vumc.org, Vice President for Knowledge Management, Director, Center for Knowledge Management, Vanderbilt University Medical Center; Professor of Biomedical Informatics and Medicine, Department of Biomedical Informatics, Vanderbilt University Medical Center; Department of Medicine, Vanderbilt University Medical Center, Nashville, TN

**Keywords:** Asynchronous learning, online learning, Systematic Reviews as Topic

## Abstract

Given the key role of systematic reviews in informing clinical decision making and guidelines, it is important for individuals to have equitable access to quality instructional materials on how to design, conduct, report, and evaluate systematic reviews. In response to this need, Vanderbilt University Medical Center's Center for Knowledge Management (CKM) created an open-access systematic review instructional video series. The educational content was created by experienced CKM information scientists, who worked together to adapt an internal training series that they had developed into a format that could be widely shared with the public. Brief videos, averaging 10 minutes in length, were created addressing essential concepts related to systematic reviews, including distinguishing between literature review types, understanding reasons for conducting a systematic review, designing a systematic review protocol, steps in conducting a systematic review, web-based tools to aid with the systematic review process, publishing a systematic review, and critically evaluating systematic reviews. Quiz questions were developed for each instructional video to allow learners to check their understanding of the material. The systematic review instructional video series launched on CKM's Scholarly Publishing Information Hub (SPI-Hub™) website in Fall 2023. From January through August 2024, there were 1,662 international accesses to the SPI-Hub™ systematic review website, representing 41 countries. Initial feedback, while primarily anecdotal, has been positive. By adapting its internal systematic review training into an online video series format suitable for asynchronous instruction, CKM has been able to widely disseminate its educational materials.

## CONTEXT, AIMS, AND SIGNIFICANCE OF PROJECT

In the Fall of 2023, the Center for Knowledge Management (CKM) at Vanderbilt University Medical Center (VUMC) released an online instructional video series on systematic reviews (https://spi-hub.app.vumc.org/sysrev/home) [1]. The project aimed to develop a freely available learning resource to ensure equitable access to quality educational materials on systematic reviews. The project further aimed to strengthen evidence-based medicine initiatives by equipping individuals with the knowledge and skills to design, conduct, and evaluate systematic reviews.

The idea for developing the online instructional video series emerged from an internal CKM training series on systematic reviews, which was conducted in Spring 2023. The training consisted of eight-hour-long sessions designed and led by three CKM team members experienced in conducting systematic reviews. The goal of the internal training was to strengthen the overall capacity of the CKM team to conduct and contribute to systematic reviews at VUMC. After the series ended, CKM team members discussed ways to share the training with other individuals and groups wanting to learn more about systematic reviews. The team ultimately chose to adapt the content developed for the internal training series into a video series format to allow for online, asynchronous instruction for an external audience.

The online systematic review training was created by CKM's team of information scientists and made available through the Scholarly Publishing Information Hub (SPIHub^™^); an open access tool developed by CKM. Although SPI-Hub^™^ was originally conceived with the intent of helping authors identify suitable journals in which to publish their work while steering them away from controversial journals, the site over time has become a truly comprehensive one-stop shop for researchers interested in learning more about the publishing industry, researcher profiles, upcoming trends on open access tools and policies, and now, through the link to the systematic reviews training, evidence provision. Additionally, as SPIHub^™^ consistently vaunts an average of 4,000 monthly national and international uses, its site was determined to be the perfect platform for the systematic review instructional videos [2].

## PROJECT DESCRIPTION AND TECHNOLOGY

The SPI-Hub™ systematic review training website includes brief instructional videos covering all aspects of the systematic review process. Topics discussed include types of literature reviews, reasons for conducting a systematic review, how to develop a systematic review protocol, steps in conducting a systematic review, web-based tools to aid with the systematic review process, publishing a systematic review, and appraising systematic reviews.

In total, 18 videos, with an average length of 10 minutes each, were created over a period of approximately four months. The instructional videos are accompanied by quiz questions to allow users to check their understanding of the material. The questions were formatted to provide feedback and allow for unlimited re-attempts. Each video page also lists learning objectives and key references. The project team included five information scientists and two application developers; the initiative was led by the Center's director.

Content within the systematic review section of SPIHub™ is delivered through Laravel, an open-source PHP Framework, in conjunction with internally developed HTML5 scripts. The instructional videos were created using the PowerPoint screen recording feature.

## ADVANTAGES, LIMITATIONS, AND IMPACT

By adapting CKM's internal systematic review training series into an online video series format, CKM has been able to reach a large and diverse audience. Usage data has been monitored to help assess the project's impact. From January through August 2024, there were 1,662 accesses to the systematic review instructional series, representing 41 countries. There was a notable peak in May, likely attributable to CKM's presentation at the Medical Library Association annual conference [3].

The CKM team has promoted the use of the SPI-Hub™ systematic review video series, including incorporating it in courses where appropriate. For example, the resource was shared with graduate students in a lecture on literature reviews for a biomedical informatics course on scientific communication. Additionally, the resource was shared with fourth-year medical students enrolled in the institution's WikiMed advanced elective.

While the SPI-Hub™ systematic review training website is fully implemented, the CKM team anticipates regularly assessing and responding to user feedback. Thus far, the feedback has been primarily anecdotal; however, all groups to whom the online systematic review instruction was presented have had an enthusiastic response, commenting on its comprehensiveness, ease of use, and the fact that it was freely available through the web.
